# Geometric road design factors affecting the risk of urban run-off crashes. A case-control study

**DOI:** 10.1371/journal.pone.0234564

**Published:** 2020-06-11

**Authors:** Patricia Álvarez, Miguel A. Fernández, Alfonso Gordaliza, Alberto Mansilla, Aquilino Molinero

**Affiliations:** 1 Escuela de Ingenierías Industriales, Universidad de Valladolid, Valladolid, Spain; 2 IMUVA – Departamento de Estadística e Investigación Operativa, Universidad de Valladolid, Valladolid, Spain; 3 Departamento de Ingeniería Mecánica, Escuela de Ingenierías Industriales, Universidad de Valladolid, Valladolid, Spain; 4 Departamento de Ingeniería Energética y Fluidomecánica, Escuela de Ingenierías Industriales, Universidad de Valladolid, Valladolid, Spain; University of British Columbia, CANADA

## Abstract

**Objective:**

Single vehicle run-off crashes in urban areas constitute a growing problem that deserves more attention from authorities and researchers. This study aims to detect geometric road design risk factors characterizing places where urban run-off crashes might happen.

**Methods:**

A case-control study was performed in the urban area of Valladolid (Spain) with data corresponding to a four-year period. Logistic regression models were used to analyze data, considering different variables related to design parameters in the models: type of intersection, radius of curvature, width of the pavement, width of the traffic lane, number of lanes for traffic in the same direction, direction of the traffic, length of the previous straight section, distance to the previous traffic light, slope, and finally, priority regulation. Two different scenarios were investigated: intersections and curves.

**Results:**

The Adjusted Odds-Ratio of a run-off crash was five times higher in double direction roads with median strip than in one-way urban roads, for both curves and intersections, and almost nine times higher on road sections with previous straight lengths greater than 500 meters. Specific risk factors for intersections are *“number of lanes for traffic in the same direction”* (the odds of a run-off crash are more than five times higher on a road with two or more lanes), *“length of preceding straight section”* (the odds on road sections with lengths greater than 500 meters are more than nine times that of road sections with a length of less than 150 meters). For curves, specific factors are *“width of the traffic lane”* (the odds of a run-off crash on curves with lanes wider than 3.75m are more than six times higher) and *“priority regulation”* (the odds of a run-off crash increases more than twelve times on road sections with traffic light regulation over those without any regulation).

**Conclusions:**

The current study identifies urban road configurations that might require redesigning with the aim of decreasing the odds of a run-off crash, or the implementation of passive protective systems to mitigate their consequences. Specifically, intersections in two direction roads with median strip, more than two lanes per direction and a long preceding straight section, as well as curves with wide lanes and traffic light regulation, are the places that require attention.

## Introduction

Although 1.35 million people die yearly on the roads, traffic crashes are predictable and preventable [1]. In most countries around the world, about 30% of all road fatalities are single vehicle crashes, [2] where, for instance, one vehicle leaves the road and strikes a rigid object, rolls over or goes down a steep slope. In the European Union, [3] a third of all traffic fatalities are run-off crashes. Although run-off crashes could be considered more prevalent on rural roads (for instance, 38% of fatalities and 36% of hospitalized injured casualties on rural roads were due to run-off road crashes [4]), run-off crashes in urban environments must also be considered due to their high frequency (for instance, in South Australia [5] and New Zealand, run-off crashes amounted to 20% of urban injury crashes and 48% of urban fatal crashes; moreover, the second most frequent type of urban injury crash was run-off road collision (16%) [4]). The increased incidence of urban run-off crashes could be related to the increased exposure to urban environments: more than 50% of the world population lives in urban areas and this share is expected to increase in the future (i.e., in Spain, up to 75% in 2050 [6]).

There is extensive literature on analyzing risk factors in crashes. Table 1 provides a (non-exhaustive) recollection of interesting contributions and findings. Different studies [7–11] have analyzed road risk factors involved in run-off crashes on rural roads, while others provide descriptive [12] or inferential [13–15] studies of run-off crashes on urban roads; although they [13–15] have sometimes focused only on very specific locations (urban arterial roadways or urban shopping road segments). Other studies [16–20, 21] provide very interesting and specialized statistical models for studying specific types of crashes such as sideswipe and rear-end crashes [16, 19], left-turn bay crashes [17], or only car-car rear-end crashes [20]. General information about run-off crashes in rural and urban areas together is also given [22,23].

**Table 1:** Literature review summary.

Scope	Scope category	Reference	Type of Study	Geometric road factors detected
Rural crashes	Run-off crashes	[7]	Inferential (Logistic regression)	Road alignment (*curves*)
		[8,9]	[8] Descriptive [9] Descriptive	Road layout:.- *Standard edge lines*, *centerlines*, *line-markings with high wet-night visibility and reflectors or panels of retroreflective sheeting on curves*‥*- Curves*.
		[10,11]	[10] Inferential (Negative binomial estimation) [11] Descriptive	Road width and layout:.*- Lane*, *median and shoulder width*, *approach width to bridges*, *roadside hazardous objects and slopes*‥*- Clearer edge and centre lines*, *rough surface*, *curves*, *lane and shoulder width*, *number of lanes*, *presence of parked cars on the road edge*, *presence of vertical elements on the roadside and sight distance*.
Urban crashes	Run-off crashes	[12]	Descriptive	No *geometric road factors* were found (only information about road surface type, condition and character).
	Run-off crashes (only on specific urban areas: urban arterial roadways [13], shopping districts areas,…[14,15])	[13–15]	[13] Inferential (Negative binomial regression model) [14] Inferential (Logistic regression) [15] Inferential (Cross-sectional analysis method)	Roadside design strategies: *livable-street treatments*, *shoulder width and unpaved fixed-object offsets*. Road alignment and layout *(road cross section (curves*, *presence of median)*, *road type*, *sharing the road with other vehicle types (trams and bicycles)*, *roadside poles*, *and local amenities)*. *Roadside environment and facilities and amenities*.
	Sideswipe-same direction and rear-end crashes	[16]	Inferential (Cross-sectional analysis method)	Road width (*lane width*)
	Left-turn bay crashes	[17]	Inferential (conflict-based and collision-based observational Before-After Studies, BA studies)	Road layout (*left-turn bay length*).
	General crashes	[18]	Inferential (Cross-sectional analysis method)	Road width (*lane width*).
Rural and urban crashes together	Rear-end, sideswipe, and angle collisions in freeway diverge areas	[19]	Inferential (random parameters multivariate Tobit (RPMV-Tobit) models))	Road geometries *(on rear-end and sideswipe crashes*: *lane-balanced design*, *number of lanes on mainline*, *and ramp length; and deceleration lane length only on rear-end crashes*. *No geometric road factors were found on angles crashes)*.
	Car-car rear-end crashes	[20]	Inferential (random parameters bivariate ordered probit models))	*No* geometric *road factors* were found. (Other type of risk factors: Driver age, gender, vehicle, airbag or seat belt use, etc.,).
	Run-off crashes	[22,23]	[22] Descriptive [23] Descriptive	Road width (*lane and shoulder width)*. Road layout and *roadway width (advisory signs*, *curves*, *and shoulders)*

To conclude, the literature analyzed has shown which geometrical road factors are related to rural run-off crashes; however, concerning run-off crashes in urban areas, the inferential studies found only deal with very specific urban areas or crash types, or they use complex statistical methods.

The study developed in this article uses a common statistical case-control design to determine which geometric road design characteristics contribute to a higher risk of a run-off collision occurring in an urban area. As a consequence, we believe that the results set out here can be considered for future well-designed and properly maintained roads. In fact, some current guides or recommendations [24, 25, 26] are already focusing on aspects that must be considered for appropriate road designs.

The study analyses data collected in the city of Valladolid over a four-year period (staring in 2009). Valladolid is a Spanish city, the capital of the province of Valladolid and headquarters of the Regional Government of *Castilla y León*. Valladolid is a mid-sized city, with around 300,000 inhabitants [27], and is the 13^th^ most populous municipality in Spain and the first in all the Spanish northwest, which means that it can be considered representative of many other cities in Spain.

In the following sections, we describe the variables and values used in this study, as well as their sources. Then, the statistical methods used are detailed and, finally, the results obtained and their practical interpretation are set out with the aim of improving the road safety of urban roads.

## Methods

For the purpose of this study, an urban road is one within the boundaries of a built-up area, which is an area with entries and exits especially sign-posted as such, and with a speed limit of 50 km/h, which is the general speed limit in Spanish cities and urban areas.

### Materials

Two databases were considered: ARENA and PIMUSSVA.

The **ARENA database** is designed and maintained by the General Directorate of Traffic, the Spanish administration in charge of managing road traffic. ARENA contains information from all injury traffic crashes on Spanish roads. This information is gathered by the national traffic police, after asking any people involved in the crash, carrying out the respective reconstructions and gathering information from and about non-injured and injured people, which means any person who was not killed, but sustained one or more serious or slight injuries as a result of the crash, as well as any people who were killed at the time of the crash or within 30 days after its occurrence. Some of the variables included in ARENA correspond to the geometric design parameters of the road where the crash occurred.

The **PIMUSSVA study** is a crash database of the city of Valladolid created by the City Council [28]. It contains data about all traffic crashes (including crashes with only material damage) that occurred in Valladolid between 2009 and 2013. This information is gathered by the local police in the city of Valladolid after receiving any notification from the emergency services. This database was considered in addition to ARENA in order to increase the number of cases in the study, because non-injury crashes are also considered. However, PIMUSSVA does not include information about geometric road parameters, so, the location of each crash within PIMUSSVA was individually analyzed to include the geometric parameters needed to perform the analysis.

Some discrepancies between both databases were found, especially in the way in which some variables were coded. Moreover, in ARENA, it is sometimes difficult to accurately find the location due to a lack of information. To overcome these difficulties, those cases that presented any doubt were individually investigated with the local police. Lastly, we would like to add that the information in these databases is considered in trials related to traffic crashes, at both national and local level. For these reasons, the data used in this study were reliable and no more calibration on our crash data has been needed.

### Variables

The variables included in this study are shown in Table 2 (Variable V1 to Variable V10) and were selected because they are geometric road design parameters. In other words, they are the parameters a technical department could consider to design a road. In our study, continuous variables were transformed into categorical variables to facilitate the statistical analysis after using objective categorization criteria [29]. In fact, the official databases dealt with the categorized variables and, provided that the observed frequencies allowed it, the boundaries between categories stated in the official databases have been respected. Note also that the categorization of variables has the advantage of allowing a non-linear relationship between the response and the predictors to be taken into account. Several possibilities were considered for this categorization and it was found that no significant differences appeared due to different categorizations.

**Table 2:** Raw odds-ratios (rOR) and their 95% CIs for the variables considered in each of the three different scenarios.

Description		Proportion		Raw Odds-Ratios (rOR)		
Variable	Category	Cases (%)	Controls (%)	Curves + Intersections	Intersections	Curves
Type of intersection (V1)	T or Y (ref)	25.6	45.1			
	X or +	14.6	23.2	1.113 (0.453,2.736)	1.113 (0.453,2.736)	-
	Roundabout	11.0	4.9	3.96** (1.087,14.456)	3.96** (1.087,14.456)	-
	Roundabout with transitional central island	25.6	3.7	12.333*** (3.285,46.305)	12.333*** (3.285,46.305)	-
	Curve	23.2	23.2	1.762 (0.767,4.046)	-	-
Radius of curvature (V2)	<50 m (ref)	13.4	7.3			
	50–100 m	4.9	3.7	0.727 (0.121,4.388)	-	0.727 (0.121,4.388)
	>100 m	4.9	12.2	0.218* (0.047,1.005)	-	0.218* (0.047,1.005)
	Intersection	76.8	76.8	0.545 (0.190,1.565)	-	-
Width of the pavement (V3)	<6 m (ref)	15.9	30.5			
	6-9m	59.8	47.6	2.416** (1.095,5.330)	3.111** (1,268,7.632)	1.000 (0.178,5.632)
	>9 m	24.4	22	2.137 (0.848,5.386)	1.820 (0.632,5.241)	3.111 (0.414,23.393)
Width of the traffic lane (V4)	< = 3.75m (ref)	57.3	61			
	>3.75 m	42.7	39	1.164 (0.624,2.170)	0.767 (0.375,1.568)	4.800** (1.204,19.129)
Number of traffic lanes for the same direction of the traffic (V5)	1 (ref)	40.2	76.8			
	2 or more	59.8	23.2	4.923*** (2.502,9.687)	8.810*** (3.836,20.234)	1.000 (0.276,3.625)
Direction of the traffic (V6)	One way (ref)	26.8	41.5			
	Double way	26.8	48.8	0.850 (0.403,1.794)	0.846 (0.351,2.040)	0.667 (0.148,3.011)
	Double with median strip	46.3	9.8	7.341*** (2.89,18.64)	14.95*** (4.486,49.83)	1.042 (0.177,6.123)
Length of the previous straight section (V7)	<150 m (ref)	8.5	28.0			
	150–500 m	45.1	54.9	2.702** (1.043,6.995)	2.971* (0.888,9.939)	2.567 (0.518,12.723)
	>500m	46.3	17.1	8.918*** (3.138,25.349)	11.00*** (3.060,39.541)	5.833 (0.696,48.873)
Distance to the previous traffic light (V8)	<150m (ref)	32.9	41.5			
	150–500 m	56.1	48.8	1.448 (0.749,2.800)	1.185 (0.550,2.552)	2.694 (0.713,10.178)
	>500 m	11.0	9.8	1.417 (0.482,4.164)	1.479 (0.466, 4.694)	-
Slope (V9)	No (ref)	87.8	93.9			
	Yes	12.2	6.1	2.139 (0.697,6.559)	1.844 (0.512,6.643)	3.375 (0.318,35.78)
Priority regulation (V10)	None (ref)	42.7	50.0			
	Stop sign	3.7	13.4	0.319* (0.082,1.237)	0.285* (0.070,1.161)	-
	Yield sign	11	20.7	0.620 (0.246,1.565)	0.553 (0.204,1.500)	-
	Traffic light	42.7	15.9	3.154** (1.445,6.881)	2.527** (1.036,6.160)	8.308* (0.890,77.568)

where

* stands for p-value < = 0.1,

** for p-value < = 0.05 and

*** for p-value< = 0.01. Sample size n = 82 (63 intersections and 19 curves) was used for both cases and controls. Notice that the “Curve” category is removed from the variable “Type of intersection” (V1) in the second scenario (only “Intersections”), and that the “Intersection” category is removed from the variable “Radius of curvature” (V2) in the third scenario (only “Curves”).

On the other hand, no variables related to speed are included in the study because the speed limit on all locations considered is always constant and equal to 50 km/h. Note that the statistical units in our study are the locations where crashes have occurred (cases) or where they have not occurred (controls), not the crashes themselves. Another variable that has not been included in the model is traffic volume. This variable is obviously not a geometric road design factor, which is our main interest here, but may be related to the number of general crashes appearing at a location [30]; although some studies have found that vehicle flow does not explain much variability of itself [31, 32, 33]. The main reason for not including this variable in our study is that there are not sufficiently accurate values available. There are measurement points in the city, but the network is not dense enough to provide accurate values. However, there are other variables in the study that can be considered as “proxy” variables for traffic volume, such as the “number of traffic lanes” or “the width of the pavement”. In this sense, we believe that at least a great part of the possible effect of traffic volume in the problem has been accounted for. We have also made a recommendation to the city council to improve the network of points measuring the traffic volume, so that in can be included in future studies.

### Study design and sample selection

This study focuses on intersections and curves, as these are the places where urban run-off impacts are more likely to happen [1, 2, 3, 4]. A case-control design was used to identify road-related risk factors associated to run-off crashes. The outcome was the occurrence of a run-off crash at either an intersection or a curve. As outcomes only appear in a small fraction of exposed and unexposed individuals, a case-control study [21] allows the magnitude of association between the different exposures considered and the outcome to be estimated in a more efficient way than other epidemiological and clinical research designs. It is important to notice that the individuals in our study are, as mentioned previously, the locations where crashes have occurred (cases) or where they have not (controls), not the crashes themselves; we can therefore use the term “sites” when we refer to the “individuals” in the study.

All run-off crashes occurring at intersections and curves of Valladolid recorded in the ARENA and PIMUSSVA databases between 2009 and 2013 were considered in the study. This represents 82 cases, 63 intersections and 19 curves.

The same amount of controls was randomly selected from all population individuals (locations or sites) that did not suffer the said outcome in the period. This random selection is not an immediate task, due to the inexistence of an organized census containing all intersections and curves in the Valladolid urban area. To perform a truly random selection, the map of the city of Valladolid was split into a regular grid of squares measuring 150 meters on each side. Then, a square from the grid and a vertex of the square were randomly chosen, and finally the intersection inside the square closest to this vertex was selected. If the intersection did not belong to the set of cases, then it was incorporated into the group of controls. If there were no intersections in the selected square, a new random selection was done. A similar procedure was used for the selection of the curves.

### Data analysis/statistical methods

Logistic regression was used to analyze these data for three different situations: curves, intersections and the combination of both. For each of these three situations, unadjusted logistic regression models served to obtain raw odds-ratios (rOR) to check which road-related variables may influence the outcome. Multivariate logistic regression, including all possible explanatory variables, was used to calculate the adjusted odds-ratios (aOR) so as to check the role of each variable in the presence of the others present in the model. Stepwise selection procedures were used to develop the logistic regression models. From these procedures, three models for each of the three abovementioned situations were selected: a full model including all variables, the model resulting from a backward selection procedure, and an intermediate model performing well from the correct classification point of view. To avoid upwards bias in the proportion of correctly classified observations, when building and assessing the model using the same sample, a leave-one-out cross-validation (LOO) procedure was used to evaluate the models. Finally, to obtain a definitive model for each of the three situations, the associated ROC curves [33] of each of the models and the De Long test [34] were used to compare the areas under the ROC curves (AUC) and select the final models. We chose AUC as the criterion for selecting from among the models, instead of others such as the Akaike Information Criterion (AIC), as AUC is more closely related to a good classification performance than AIC, which is a more general criterion, mainly dealing with model fitting. All these analyses were performed with IBM SPSS software (Armonk, NY, USA) for model estimation and variable selection and SAS software (Cary, NC, USA) for the LOO evaluation and ROC curves tests.

## Results

### Descriptive analysis

Table 2 shows the frequency distribution (in percentages) of all variables considered in the study for both controls and cases.

### Analytical study

The rOR were obtained by studying the effect of each single predictor variable in the logistic regression models. They are provided in Table 2.

The variables Type of intersection (V1, categories “Roundabout with transitional central island” and “Roundabout”), Width of the pavement (V3, category “6–9 m”), Number of traffic lanes for the same direction of traffic (V5), Direction of the traffic (V6, category “Double with median strip”) and Length of the previous straight section (V7, category “>500 m”) all appear highly significant in the regression models of both Curves+Intersections and Intersections (p<0.05 or even p<0.01); whereas they do not for the Curves model. The variable Width of the traffic lane (V4) appears to be significant (p<0.05) only for the Curves model. The remaining variables or categories appear to be less significant, with p>0.05.

It is important to note that most of the significant variables in the rOR analysis also appear in the final selected models, so the corresponding aOR are also (reasonably) significant. The most important changes from rOR to aOR are those appearing for Radius of curvature (V2), both in the Curves+Intersections and Curves multiple logistic regression models, and those appearing for Direction of the traffic (V6) and Priority regulation (V10) in the Curves model.

As for the adjusted models, Table 3 shows the results corresponding to the analysis of the ROC curves and the LOO values for the three models considered for the three situations (intersections + curves, intersections only and curves only).

**Table 3:** Model selection for the three scenarios (Intermediate model was selected as “reference category”).

Data	Model	Variables in model	LOO	AUC	p-value
Scenario “Curves and Intersections”	Full	All	67.7%	0.8484	0.1068
	Intermediate	V1, V2, V5, V6, V7	70.1%	0.8214	0.1671
	**Backward**	**V1, V2, V6, V7**	**68.9%**	**0.8105**	
Scenario “Only Intersections”	Full	All (except V2)	70.6%	0.8816	0.4214
	Intermediate	V1, V3, V5, V6, V7, V8	73%	0.8731	0.1337
	**Backward**	**V3, V5, V6, V7**	**73%**	**0.8490**	
Scenario “Only Curves”	Full	All (except V1)	63.2%	0.9488	0.0566
	**Intermediate**	**V2, V4, V6, V10**	**78.9%**	**0.8753**	**0.0578**
	Backward	V4, V10	73.7%	0.7701	

In the case of considering intersections and curves together, the backward model was chosen over the other two possibilities. The full model has a lower LOO value than the intermediate and backwards model while the AUC value of the intermediate model is not significantly higher than that of the backwards model (p-value 0.2812). Table 4 shows that, for the chosen model, curves had significantly higher odds (aOR = 5.664) than the reference class (Type of intersection (V1): T or Y intersections), while there were no significant difference in the odds between this reference class and the other types of intersections. For the radius of curvature (V2), a significant decrease in odds appears when the radius of curvature was increased (aOR = 0.061). The Direction of the traffic (V6) had a significant role, with double direction roads with median strip showing increased odds (the odds of a run-off crash on a double direction road with median strip are more than five times that of a crash on a road with a single lane) over single direction roads. Double direction roads showed a slightly (non-significant even at 0.1 level) decrease in the odds, but the inclusion of the median strip on these roads modified the result to a significant odds increase. An increase in the length of the preceding straight section (V7) resulted in a significant increase of odds. When the length was between 150 and 500 meters, the odds of a crash on a road with this length are more than three times that of a crash on a road with a previous straight section of less than 150 meters in length, and almost nine times when the length is over 500 meters.

**Table 4:** Final models selected for each scenario.

	Variable	Category	ß	Exp (ß) (aOR)	95% CI
Scenario “Curves and Intersections”	Constant		-1.745***	0.175	
	Type of intersection (V1)*	T or Y (ref)			
		X or +	0.207	1.230	(0.454,3.332)
		Rotary	1.020	2.772	(0.583,13.172)
		Roundabout with transitional central island	0.777	2.175	(0.451,10.495)
		Curve	1.734***	5.664	(1.577,20.334)
	Radius of curvature (V2)**	<50m			
		50-100m	-0.585	0.557	(0.079,3.929)
		>100m	-2.799***	0.061	(0.009,0.413)
	Direction of the traffic (V6)***	Single (ref)			
		Double	-0.751	0.472	(0.192,1.158)
		Double with median strip	1.649***	5.201	(1.593,16.982)
	Length of the previous straight section (V7)***	<150m (ref)			
		150-500m	1.165 **	3.206	(1.065,9.650)
		>500m	2.196 ***	8.985	(2.490,32.421)
	Hosmer-Lemershow GoF test χ^2^ = 4.913, df = 8, p-val = 0.767				
Scenario “Only Intersections”	Constant		-2.108***	0.121	
	Width of the pavement (V3)**	<6m (ref)			
		6-9m	0.374	1.453	(0.437,4.835)
		>9m	-1.368	0.255	(0.045,1.439)
	Number of traffic lanes for the same direction of the traffic (V5)**	1 (ref)			
		2 or more	1.665 **	5.283	(1.216,22.953)
	Direction of the traffic (V6)*	Single (ref)			
		Double	-0.328	0.720	(0.220,2.358)
		Double with median strip	1.606 **	4.981	(1.133,21.906)
	Length of the previous straight section (V7)**	<150m (ref)			
		150-500m	1.209 *	3.349	(0.793,14.138)
		>500m	2.225 ***	9.250	(1.934,44.247)
	Hosmer-Lemershow GoF test χ^2^ = 6.107, df = 7, p-val = 0.527				
Scenario “Only Curves”	Constant		-1.220 **	0.295	
	Radius of curvature (V2)	<50m			
		50-100m	-0.660	0.517	(0.034,7.785)
		>100m	-2.463 *	0.085	(0.007,1.024)
	Width of the traffic lane (V4)**	< = 3.75m (ref)			
		>3.75m	1.848 **	6.346	(1.376,29.274)
	Direction of the traffic (V6)	Single (ref)			
		Double	-0.406	0.666	(0.086,5.171)
		Double with median strip	2.700	14.879	(0.555,398.61)
	Priority regulation (V10)**	None (ref)			
		Traffic light	2.496 **	12.13	(1.112,132.36)
	Hosmer-Lemershow GoF test χ^2^ = 4.527, df = 8, p-val = 0.883				

where

* stands for p-value < = 0.1,

** for p-value < = 0.05 and

*** for p-value< = 0.01.

Focusing only on intersections, the analysis included in Table 3 suggested that the backward model was the most appropriate, as its LOO value is equal and its AUC value is not significantly lower than that of the intermediate model. In the intersections only analysis, the variable Radius of curvature (V2) was directly dropped from the model and the category Curve was removed from the variable type of intersection (V1). Regardless of this, the variable V1 was also dropped from the model during the variable selection procedure. The Direction of traffic (V6) and the Length of previous straight section (V7) play the same role as in the global case (intersections and curves together). The variables Number of traffic lanes for the same direction of traffic (V5) and Width of pavement (V3) were included in the selected model. An increase in the number of traffic lanes significantly increased the odds in the intersections: the odds of a run-off crash on a road with 2 or more lanes are more than five times that of a crash on a road with a single lane. The interpretation of the influence of V3 (Width of pavement) is more complicated. V3 was significant, although there were no significant differences between the reference class (<6m width) and the other two classes. An inspection of the odds associated to the other two categories of V3 showed that the odds associated to the reference class are in between the other two. By default, the lowest values of the originally numerical variables were chosen as the reference when they were transformed into categorical variables. The lowest odds are found when the width is highest (>9m) and the highest odds when the width is intermediate (6-9m).

The third scenario is only curves. As with the previous analysis, the variable Type of intersection (V1) was dropped directly. Table 3 shows that the p-values of both AUC tests were on the border of rejection, considering the usual 0.05 cut-off point. Thus, the full model was close to achieving a significant difference with respect to the intermediate model, and the same happens to the intermediate model with respect to the backward model. In addition, the intermediate model exhibited a higher LOO value than either the full or the backward models. Therefore, the intermediate model was chosen in this third analysis. The resulting model had two common variables with the model chosen for intersections and curves considered together: Radius of curvature (V2) and Direction of traffic (V6), which are not globally significant at the 0.05 level, but increased the LOO predictive ability of the model from 73.7% to 78.9% (see Table 3). The influence of V2 and V6 were similar in both analyses. The variable V4, Width of traffic lane, indicates that the odds of a crash on a road with the lane width higher than 3.75m are more than six times the odds of a road with less than 3.75 meters lane width. The variable V10 (Priority regulation) suggests that the odds of a crash on a road section with traffic light regulation is more than twelve times the odds of a crash on a road without any regulation.

In order to check the goodness of fit for these regressions, we considered the Hosmer-Lemeshow (HL) goodness of fit test. The results for these HL tests are given in Table 4 and the large p-values suggest that the models are appropriate.

To summarize the results, we can say that urban locations with a potentially higher probability of inducing run-off crashes are: *Curves* when the “width of traffic lane” is more than 3.75 meters, (the odds of a run-off crash on curves with lanes wider than 3.75m are more than six times higher) or when “there are traffic lights” to regulate the priority (the odds are more than twelve time higher), and *Intersections* when the “number of traffic lanes for the same direction of traffic” is 2 or more lanes (the odds of a run-off crash with two or more lanes are more than five times those of a road with a single lane), when the “direction of the traffic” is double with a median strip (the odds are almost five times those of single roads), and finally when the “length of the previous straight section” is higher than 500 meters (the odds on road sections with lengths greater than 500m are more than nine times those of road sections with a length of less than 150 meters).

## Discussion

Single vehicle run-off crashes in urban areas constitute a growing problem that deserves more attention from authorities and researchers. This study focuses on detecting geometric features characterizing places where urban run-off crashes can happen, with the aim of providing an effective tool for traffic authorities to recognize the places where preventive and passive safety countermeasures (for instance, installing road restraint systems, one of the most efficient road infrastructure solutions [35]) should be applied.

The study addresses intersections and curves, since these are the places where urban run-off impacts are most likely to happen. Logistic regression models have been used to analyze data in three different scenarios: curves, intersections and curves plus intersections combined.

Both scenarios (curves plus intersections combined) share the variable “direction of the traffic” as statistically significant with the category “double with median strip” notably increasing the risk of crashing, as well as the variable “length of the previous straight section” (lengths greater than 500 meters). Nevertheless, since variables separating curves and intersections (“type of intersection” and “radius of curvature”) appear as statistically significant, the model adjusted for the whole scenario shows the need to consider separate models for curves and intersections.

In the model for intersections only, the variables “direction of the traffic”, “number of traffic lanes for the same direction of traffic” and “length of the previous straight section” appear as significant. To understand the significance of all these risk factors, it should be emphasized that “speed in excess” could be the real underlying risk factor. On the other hand, it is reasonable to think that travelling in very long, straight sections with several traffic lanes for the same direction is a circumstance that can increase the risk of suffering a run-off crash, especially when the lanes are narrow.

In the model for curves, “width of the traffic lane” and “priority regulation” appear as specific factors. In curves where the priority is regulated by traffic lights, it has been proved that the risk of suffering a run-off is higher than when the priority is regulated by a stop sign. This does not necessarily mean that the problem is the traffic lights. We rather think that the problem comes from road users not respecting the traffic lights and increasing their travelling speed before the green light turns to red (when the driver sees the amber, she/he tends to drive faster, but is not able to control the vehicle in the curve).

As a brief summary of the results, we can say that the urban locations with a higher probability of suffering a run-off crash are: Curves when “width of traffic lane” is more than 3.75 meters or when “there are traffic lights” to regulate the priority, and intersections where the “number of traffic lanes for the same direction of traffic” is 2 or more, when the “direction of the traffic” is double direction with median strips or when the “length of the previous straight section” is higher than 500 meters. These conclusions will be especially useful for urban road administrations, as they can help them to develop new urban traffic management plans and to design ways to improve road crash statistics.

Lastly, we would like to comment that some City Councils are already deciding to apply countermeasures to reduce or mitigate urban run-off crashes, as is the case of the city of Valladolid. In this city, the Council has decided to install a suitable Urban Road Restraint System able to mitigate the consequences of these crashes in one location (main bridge street) where its geometric road parameters meet with the results of this study (a multi-lane curve with a 4 meter width lane, and where there are traffic lights before the curve to regulate the priority).

### Limitations

Although the study was focused only on the city of Valladolid during that period in which the PIMUSSVA study was carried out (2009–2013), it would be interesting to extend this study to other Spanish cities in order to increase the number of cases-controls (sample size), and therefore the representativeness of the results obtained. The main problem for this extension would surely be the limitations or complete absence of appropriate data from City Councils.

As we have already commented above, obtaining data on other variables related to traffic intensity, such as, for example, the Average Daily Traffic (ADT), might also be interesting. Although this sort of variable has sometimes been found as not significant in run-off crash studies [31, 32, 33], it may play a role in the appearance of general crashes, and its inclusion might assist a more accurate evaluation of the effects of the rest of the variables in the model. Nevertheless, it has been considered that this variable might also be helpful in giving priority to the implementation of safety countermeasures.

## Conclusions

One way of reducing the effects of urban crashes is to detect sites where new run-off crashes can happen and to prevent this kind of crashes (using preventive countermeasures) or to mitigate the consequences (installing road restraint systems in these locations).

There is already some literature studying which geometrical road factors are related to rural run-off crashes, but there are no inferential studies for general urban areas. Using a statistical inferential data analysis under a case-control framework instead of a descriptive study, this article has detailed the geometrical risk factors characterizing the main scenarios where an urban run-off crash is likely to happen. These conclusions are especially useful for urban road administrations, as they can help them to develop new urban traffic management plans and to design ways to improve urban road crash statistics.

## Supporting information

S1 Data (ZIP)
